# Coronary Computed Tomography Angiography-Derived Fractional Flow Reserve: A Comprehensive Review

**DOI:** 10.31083/RCM39717

**Published:** 2025-09-26

**Authors:** Dongna Yi, Fan Zhou, Quan Liang, Tongyuan Liu, Xueqin Bao, Jun Cai, Chunxiang Tang, Longjiang Zhang

**Affiliations:** ^1^Department of Radiology, Jinling Hospital, Affiliated Hospital of Medical School, Nanjing University, 210002 Nanjing, Jiangsu, China; ^2^Department of Radiology, Jinling Hospital, Nanjing Medical University, 210002 Nanjing, Jiangsu, China

**Keywords:** coronary computed tomography angiography, fractional flow reserve, coronary artery disease

## Abstract

Coronary computed tomography angiography (CCTA)-derived fractional flow reserve (CT-FFR) represents a significant technical advancement in the non-invasive evaluation of coronary artery disease, propelling CCTA into a new era of functional assessment. This review offers a comprehensive perspective on CT-FFR technology and its applications, encompassing technical refinements, diagnostic performance, indications, and other advantages. Furthermore, the implications of China-developed CT-FFR on the community and in different markets are discussed.

## 1. Introduction

Fractional flow reserve (FFR) during invasive coronary angiography (ICA) has traditionally been the gold standard for evaluating anatomical and physiological relevance in coronary artery disease (CAD). It can detect functional ischemia, guide revascularization, and predict clinical outcomes [1, 2]. However, the invasive nature, high cost, and potential complications of FFR limit its widespread use, highlighting the need for non-invasive, safer, and more cost-effective alternatives.

Recently, non-invasive FFR derived from coronary computed tomography angiography (CT-FFR) has been developed to provide both anatomical and physiological data without extra radiation exposure or stress-inducing drug use. The premier CT-FFR algorithm developed by HeartFlow® (Redwood City, CA, USA) has been undergone validation in numerous multicenter clinical trials [3, 4, 5] and has the ability to accurately identify hemodynamically significant CAD, reduce ICA use, and guide the appropriate management of CAD patients [6]. However, HeartFlow® CT-FFR requires the transfer of coronary computed tomography angiography (CCTA) data to a core laboratory supercomputer for further image processing [7]. This off-site model is expensive, time-consuming, and not readily available, thus severely limiting its use in China as well as in many underdeveloped countries.

Consequently, there is an urgent need for “on-site” CT-FFR algorithms. Significant progress has recently been made in developing these algorithms, with several domestically developed systems receiving key regulatory approvals in China: DEEPVESSEL-FFR (Keya Medical, Shenzhen, Guangdong, China; FDA/CE/NMPA), AccuFFRct (ArteryFlow Technology, Hangzhou, Zhejiang, China; CE/NMPA), skCT-FFR (ShuKun Technology, Beijing, China; NMPA), and RuiXin CT-FFR (Raysight Medical, Shenzhen, Guangdong, China; CE/NMPA) (**Supplementary Fig. 1**). Such recognition by the regulatory authorities validates performance standards and increases availability and cost-effectiveness in clinical workflows. The application of such on-site CT-FFR algorithms highlights the value of local innovation in improving the availability, efficiency, and cost-effectiveness of CT-FFR. The resulting improvement in patient management has major relevance for other countries and for the community in general. Notably, current on-site CT-FFR software in China can fully automatically provide reliable results within 4 minutes and at a lower cost for stable chest pain patients [8].

This review synthesizes recent advancements in CT-FFR, encompassing technical refinements, diagnostic validation, clinical indications, and additional advantages. Special attention is given to the impact of domestically developed Chinese CT-FFR on clinical practice and global markets.

## 2. Development of CT-FFR Algorithm

The present CT-FFR software is primarily categorized into four groups based on the methods used to calculate FFR: full-order computational fluid dynamics (CFD)-based CT-FFR, which uses comprehensive CFD for high accuracy but is computationally intensive; reduced-order CFD-based CT-FFR, which simplifies the model to reduce computational load while maintaining good accuracy; machine learning (ML)-based CT-FFR, which utilizes algorithms to learn from large datasets for efficient and faster predictions; and deep learning (DL)-based CT-FFR, which employs deep neural networks for both automated coronary artery segmentation and FFR calculation. Several prominent CT-FFR software programs are available and approved by regulatory agency in China, including semi-automatic CT-FFR tools and on-site CT-FFR technique characterized by full automation [8, 9, 10, 11, 12, 13, 14, 15, 16].

### 2.1 CFD-based CT-FFR

The implementation of CFD-based CT-FFR involves 5 fundamental steps: (1) construction of patient-specific coronary anatomical models from CCTA images; (2) estimation of baseline coronary flow with the presumption of normal supply vessels; (3) evaluation of baseline myocardial microcirculatory resistance; (4) measurement of coronary resistance changes during hyperemia; and (5) numerical solution of the Navier-Stokes equations via finite element analysis and CFD methods to derive coronary hemodynamics (flow, pressure, velocity) in both physiologic states [17]. Since the introduction of HeartFlow® CT-FFR in 2011, researchers have continued to refine computational speed and efficiency [18, 19]. Typically, one on-site CT-FFR technique employs the transluminal attenuation gradient for setting outlet boundary conditions [9]. This enables simultaneous standardization of measurements for CT instantaneous wave-free ratio and wall shear stress, thereby offering a comprehensive tool for detailed analysis of the hemodynamic mechanism in CAD. A recent multi-dimensional CFD framework, incorporating 3D (for coronary simulation), 1D (for iterative optimization), and 0D (for boundary condition definition) vascular models, has been developed to improve CT-FFR prediction accuracy and efficiency [20]. By utilizing patient-specific 0D boundary conditions and initial conditions derived from the 3D model, this framework achieves accurate and efficient CT-FFR computation.

### 2.2 ML- and DL-based CT-FFR

**Fig. 1.**
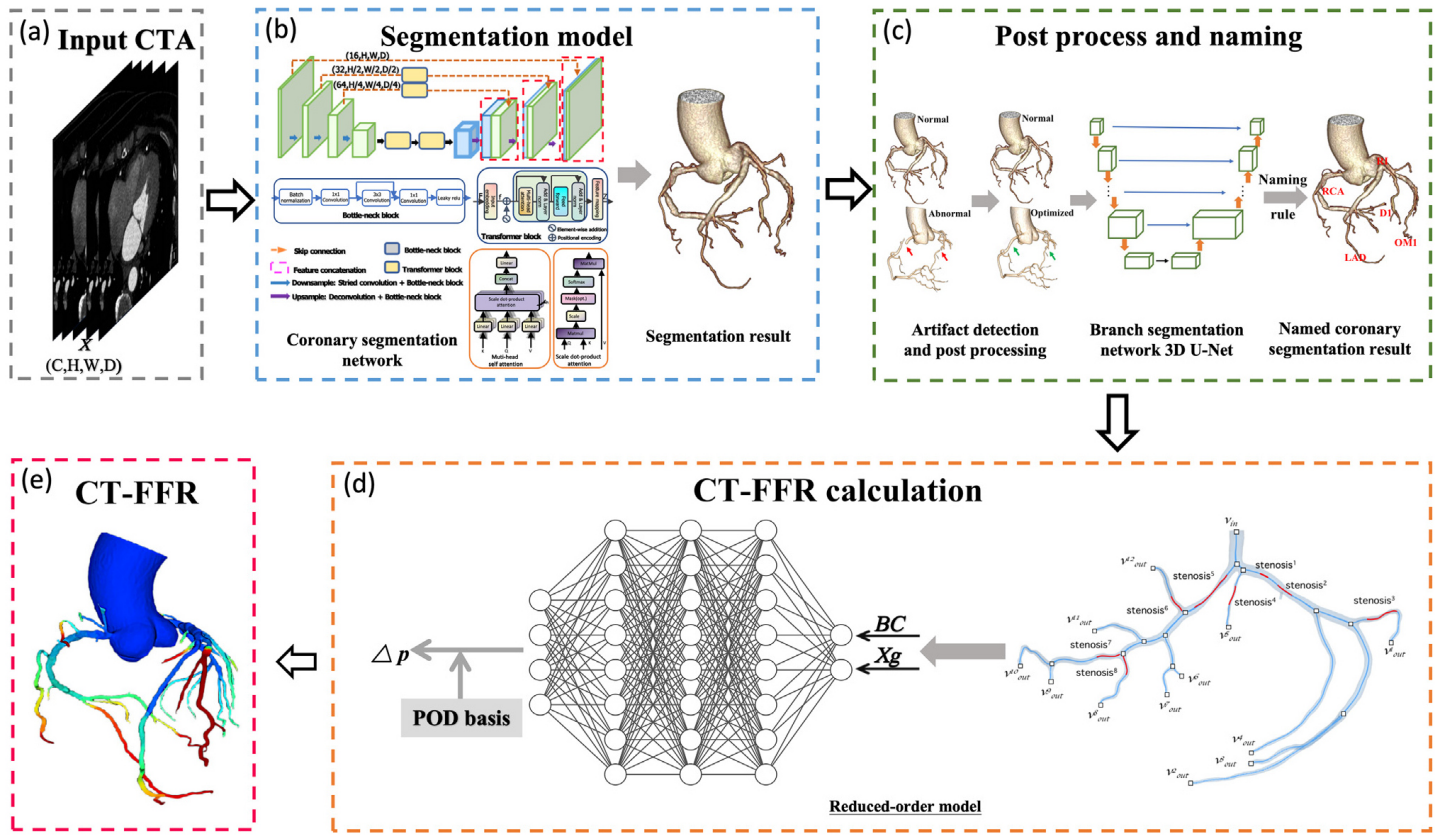
**Workflow for fully automatic ML-based CT-FFR calculation**. (a) CCTA images are used as the initial input. (b) An artificial intelligence-based segmentation model reconstructs the coronary artery tree in 3D. (c) Segmentation features of the reconstructed tree are extracted to compute CT-FFR values. (d) Regional blood flow, in both non-stenotic and stenotic segments, is reconstructed using POD coefficients and basis, allowing calculation of the pressure drop across lesions. (e) A neural network infers the POD coefficients by integrating boundary conditions (BC) and compressed Xg that represent the vessel morphology of stenotic and non-stenotic regions. CTA, computed tomographic angiography; BC, boundary conditions; Xg, shape features; POD, proper orthogonal decomposition; CT-FFR, coronary computed tomography angiography-derived fractional flow reserve; ML, machine learning; LAD, left anterior descending artery; RCA, right coronary artery; RI, ramus intermedius; OM1, first obtuse marginal branch; D1, first diagonal branch; 3D, three-dimensional. (Reprinted from Guo *et al*. [8] with permission from Elsevier).

ML-based CT-FFR methodologies have emerged as innovative approaches in functional coronary assessment. A representative ML-based technique, cFFR (Siemens Healthineers, Erlangen, Germany), employs deep learning models for offline training to extract coronary artery tree features related to hemodynamics, linking pressure distribution to patient-specific cardiovascular structures. During online prediction, it calculates the CT-FFR for individual patients using the trained model. Advanced neural networks, such as Deep Bidirectional Long-Term Recurrent Neural Network, are critical for the prediction of CT-FFR values across the coronary arterial tree [11]. The architecture operates in two sequential phases. Phase 1 employs a multilevel neural network with three fully connected layers that extract features from coronary arterial tree reconstructions, including lesion characteristics and proximal/distal markers. Phase 2 utilizes a bidirectional recurrent neural network to process feature sequences bidirectionally, incorporating contextual information from surrounding nodes through recurrent connections with learned weights. The system outputs vessel-specific FFR values, which are trained against ground truth derived from Navier-Stokes equations referenced to ICA-FFR and optimized via stochastic gradient descent. Another innovation combines an automated model for coronary plaque segmentation and luminal extraction with reduced-order 3D CFD into a fully-automated on-site CT-FFR system (Fig. 1) [8]. Employing deep learning, this technique automates coronary segmentation and establishes the CT-FFR to invasive FFR relationship, substantially cutting radiologist post-processing time and mitigating subjective bias. The resulting workflow enables end-to-end CT-FFR assessment—from CCTA image processing and report generation to stenosis quantification and ischemia evaluation—without manual intervention. With calculation times under 4 minutes and a very low rejection rate (0.2%, 1 of 464), this automated platform has been widely adopted by Chinese healthcare institutions.

## 3. Diagnostic Performance of CT-FFR and Influencing Factors 

Extensive research confirms that CFD-, ML- and DL-based CT-FFR outperform conventional CCTA in detecting lesion-specific ischemia [3, 4, 5, 8, 9, 10, 11, 13, 14, 15, 16, 21, 22, 23, 24, 25, 26, 27, 28, 29, 30, 31]. Some representative results for different CT-FFR algorithms are summarized in **Supplementary Tables 1,2,3**. Using invasive FFR as a gold standard, a recent study demonstrated superior diagnostic accuracy of fully automated on-site CT-FFR versus CCTA and ICA in detecting lesion-specific ischemia, both on a per-patient (area under the curve [AUC]: 0.82 vs. 0.55 vs. 0.57, *p* < 0.001) and per-vessel basis (AUC: 0.81 vs. 0.56 vs. 0.59, *p* < 0.001) [8]. Furthermore, a novel CFD algorithm with optimized parameters was developed, which constructs anatomic models using deep learning and subsequently computes CT-FFR under optimized boundary conditions. In gray zone lesions (FFR: 0.75 to 0.80), CT-FFR achieved 80% diagnostic accuracy, with these lesions showing less severe stenosis than FFR <0.76 lesions, but a heavier plaque burden than non-ischemic lesions [16]. Combining on-site CT-FFR with CCTA offers a diagnostic accuracy that is comparable to integrating stress CT myocardial perfusion with CCTA [27, 31, 32]. However, CT-FFR avoids additional scanning-related radiation, contrast media, and stress-inducing drugs, making it safer and potentially more widely available in clinical practice than CT perfusion.

Extensive research has examined factors influencing the diagnostic performance of CT-FFR, including patient-related, CT-FFR-related and CCTA-related factors (listed in Table 1, Ref. [21, 33, 34, 35, 36, 37, 38, 39, 40, 41, 42, 43, 44, 45, 46, 47]). The consistent diagnostic capability of CT-FFR across diverse patient subgroups highlights its utility for evaluating CAD in various clinical situations [33, 34, 35, 36, 37, 38, 39]. Among CT-FFR-related factors, the location of the measurement site is crucial, with 2 cm distal offset from the lesion terminus recommended. Of the CCTA-related factors, image quality and calcium burden are the two most important factors. Higher image quality (≥3) yields more accurate CT-FFR values. Emerging evidence suggested that diagnostic capability of CT-FFR was not significantly compromised by coronary calcification pattern, calcification remodeling index, and coronary artery calcium score (CACS) [40, 41]. While ML-based CT-FFR outperformed CT-FFR in diagnostic performance across all strata for the Agatston score, its accuracy progressively declines with increasing calcium burden [42]. These findings indicate that high calcium plaque burden may degrade CT-FFR computational accuracy. Novel photon-counting detector CT (PCCT), however, shows promise in addressing this limitation. PCCT demonstrates superior capabilities in reducing calcium blooming artifacts compared to conventional energy-integrating detector CT, especially when utilizing high-energy virtual monoenergetic imaging or ultra-high spatial resolution scanning protocols, as validated in both phantom studies and cadaveric specimens [48]. Despite PCCT having potential advantages in image quality, recent intra-individual comparative analyses reveal comparable diagnostic accuracy between PCCT-derived FFR and conventional CT systems [49, 50]. Given the high cost of PCCT and the effectiveness of existing solutions such as stress CT perfusion in handling calcium-related challenges, further studies must establish if PCCT delivers incremental value in CT-FFR assessment.

**Table 1.** **Influencing factors and clinical evidence of CT-FFR diagnostic performance**.

Factors	Evidence
Patient-related factors	Factors such as diabetes mellitus, hypertension, left ventricular diastolic dysfunction and myocardial bridge do not significantly impact CT-FFR diagnostic performance [33, 34]
	Although the myocardial bridge alters coronary anatomy during systole and affects CT-FFR values [35, 36], the morphometric characteristics (depth/length) demonstrated no significant association with CT-FFR’s predictive capability for hemodynamically significant lesions [37]
	CT-FFR has shown reliable diagnostic accuracy across patient groups with varying degrees of stenosis [38, 39]
CT-FFR-related factors	
CT-FFR algorithm	The ML-based and CFD-based CT-FFR algorithms demonstrate comparable diagnostic performance [21]
Measurement location	The optimal CT-FFR measurement requires positioning at a two-centimeter distal offset from the stenotic segment [43]
CCTA-related factors	
Reconstruction algorithm	The motion correction algorithms significantly improve CT-FFR’s diagnostic accuracy [44]
Reconstruction technology	CT-FFR measurements exhibit stability irrespective of the reconstruction approach employed, whether utilizing single-phase or multiphase cardiac data [45]
Image quality	CT-FFR diagnostic performance improves with a heart rate of <70 bpm during scanning, intracoronary enhancement within the range of 300–400 HU, and high-quality images [46]
Coronary calcification	Factors such as the coronary calcification pattern, calcification remodeling index, and coronary artery calcium score have no significant impact on the diagnostic efficacy of ML-based CT-FFR [40, 41]
	CT-FFR outperforms CCTA alone in diagnostic performance across all categories of the Agatston score [42]
	A calcium arc of >180 degrees is the sole independent predictor of incorrect diagnoses for ischemic coronary artery stenosis [47]

CCTA, coronary computed tomography angiography; CT-FFR, coronary computed tomography angiography-derived fractional flow reserve; ML, machine learning; CFD, computational fluid dynamics.

## 4. Clinical Indications for CT-FFR

The evaluation of lesion-specific ischemia is the primary application for CT-FFR in current clinical practice. According to the expert consensus from the Chinese Society of Radiology [51], CT-FFR is primarily indicated for individuals exhibiting 30% to 90% coronary stenosis on CCTA, excluding those with complex lesions.

CT-FFR applications have recently been expanded to include patients with anomalous coronary origin [52, 53], myocardial bridge (MB) [35, 36, 37, 54], and revascularization [55, 56, 57, 58] (Table 2, Ref. [35, 36, 37, 52, 54, 55, 56, 57, 58, 59, 60, 61]). CT-FFR quantification of proximal left anterior descending MB plaque progression has been established as a robust imaging biomarker [54]. Furthermore, its application has proven valuable in guiding therapeutic approaches for anomalous origin of the left coronary artery from the right sinus of Valsalva [53], and in assisting the planning phase of coronary artery bypass grafting (CABG) [56].

**Table 2.** **Expansion of clinical indications for CT-FFR and the clinical evidence**.

Indications		Evidence
Patients with suspected CAD		CT-FFR application reduces unnecessary ICA referrals and procedures revealing non-obstructive CAD, thereby improving the revascularization-to-ICA ratio [59, 60, 61]
Patients with congenital coronary artery anomalies	Patients with R-ACAOS	CT-FFR serves as a clinically relevant tool for functional assessment in R-ACAOS with interarterial course, effectively linking high-risk anatomical features to ischemic risk and angina symptoms [52]
	Patients with MB	CT-FFR demonstrates high sensitivity and negative predictive value for identifying MB-related ischemia, enabling reliable exclusion of hemodynamically relevant MB within clinical workflows [36]
		Abnormal CT-FFR values are positively associated with typical angina symptoms [35]
		CT-FFR exhibits excellent diagnostic capability for the detection of MB-related ischemia, unaffected by the depth classification of the MB [37]
		CT-FFR facilitates early intervention targeting plaque development within the proximal LAD with MB [54]
Patients before/after revascularization	Patients with stent implantation	CT-FFR can help to identify in-stent restenosis after stent placement [55]
	Patients with pre-CABG	Preoperative CT-FFR can predict 1-year graft patency and outperforms intraoperative transit-time flow measurement [56]
	Patients with pre-TAVR	Pre-TAVR CT-FFR enhances physiological assessment of coronary stenosis and reduces unnecessary ICA [57, 58]

CT-FFR, coronary computed tomography angiography-derived fractional flow reserve; CAD, coronary artery disease; ICA, Invasive coronary angiography; R-ACAOS, anomalous origin of the right coronary artery from the left coronary sinus; MB, myocardial bridge; LAD, left anterior descending artery; CABG, coronary artery bypass grafting; TAVR, transcatheter aortic valve replacement.

## 5. Guidance for Clinical Decision Making

Integrating CCTA and CT-FFR provides comprehensive coronary artery assessment, offering both anatomical and functional insights that guide treatment strategies for suspected CAD and hence reducing the rate of unnecessary ICA [59, 60, 61]. This evolution in coronary artery assessment has prompted the development and refinement of various scoring systems, transitioning from purely anatomical evaluation to integrated functional assessment.

**Fig. 2.**
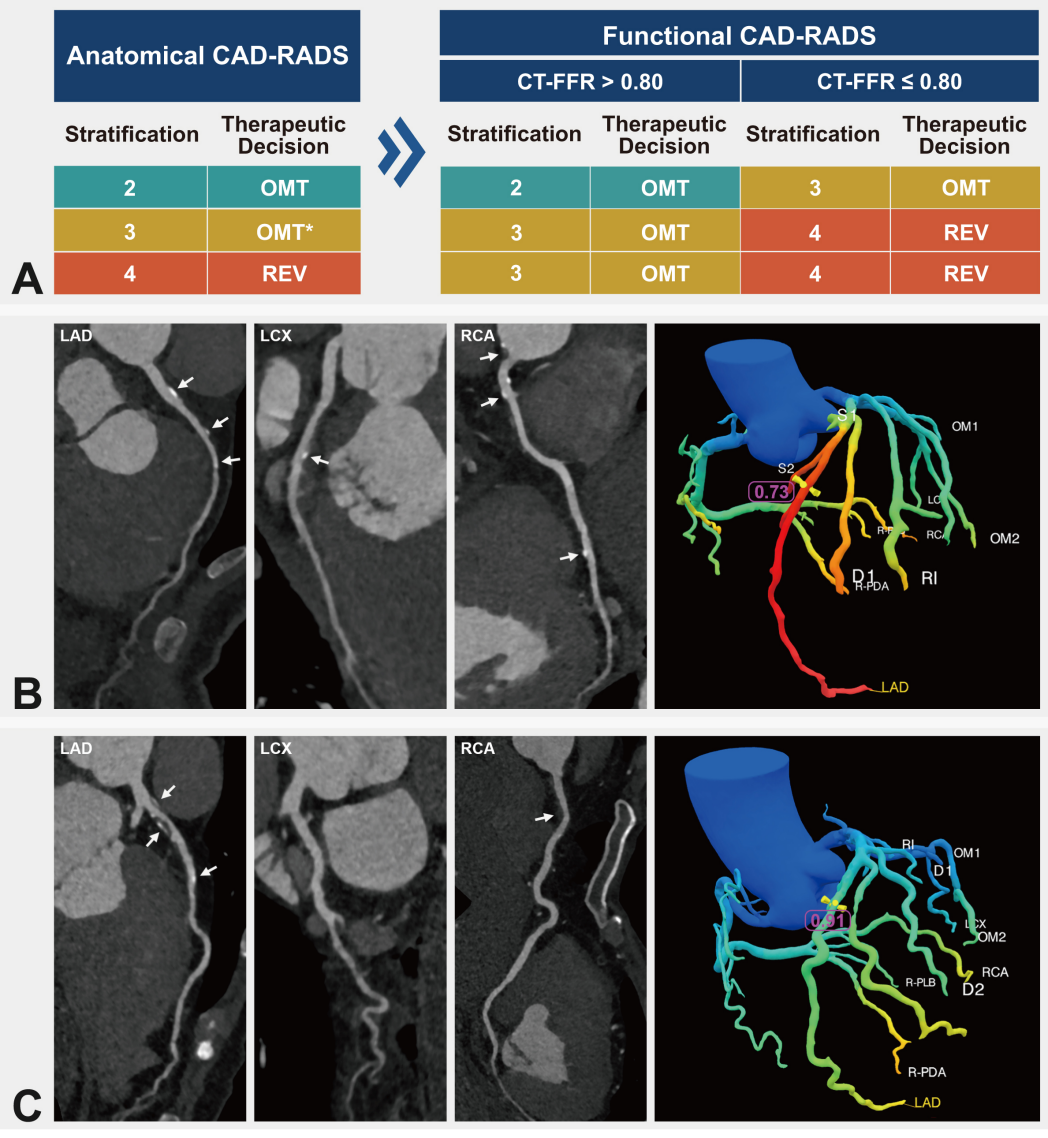
**Functional CAD-RADS scoring criteria and case illustrations**. (A) CT-FFR-based functional CAD-RADS scoring system. (B) Case illustration. A 52-year-old male presenting with 25% stenosis in the mid-segment of the LAD was classified as CAD-RADS 2. The lesion-specific CT-FFR value was 0.73, leading to a functional CAD-RADS classification of 3. (C) Case illustration. A 70-year-old male with 80% stenosis in the pro-segment of the LAD was classified as CAD-RADS 4. The lesion-specific CT-FFR value was 0.91, leading to a functional CAD-RADS classification of 3. CAD-RADS, Coronary Artery Disease-Reporting and Data System; CT-FFR, coronary computed tomography angiography-derived fractional flow reserve; OMT, optimal medical therapy; REV, revascularization; LAD, left anterior descending artery; LCX, left circumflex artery; RCA, right coronary artery. The white arrows indicate the locations of atherosclerotic plaques in the coronary arteries. * Indicates medical therapy in the category of anatomical CAD-RADS 3, whereas intensive medical therapy is usually suggested for functional CAD-RADS 3 lesions.

**Fig. 3.**
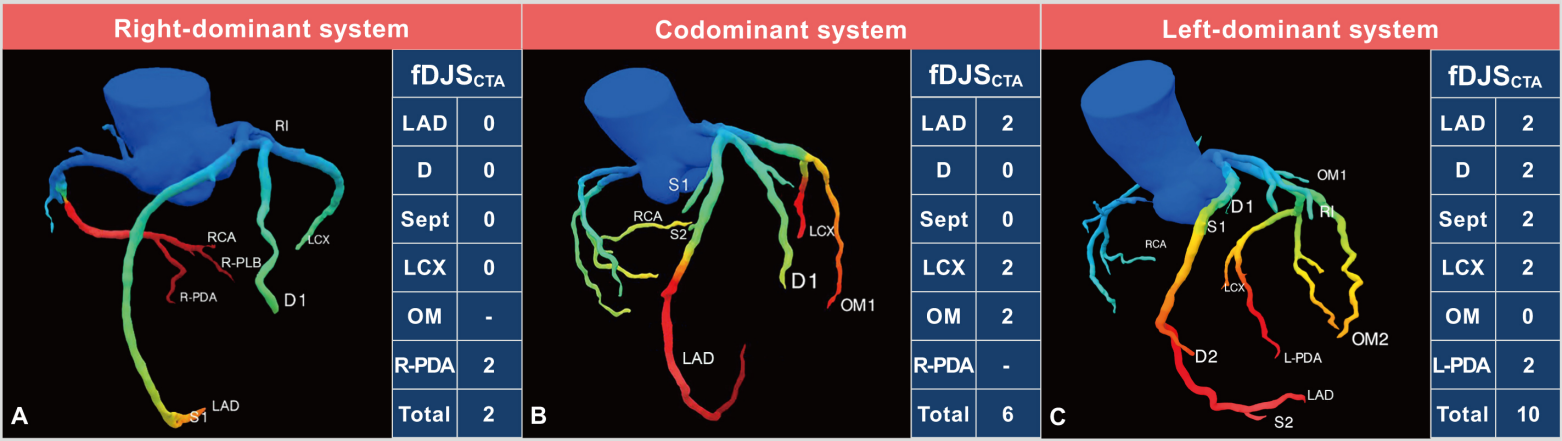
**CT-FFR based functional Duke Jeopardy Score system**. (A) Right coronary artery dominance. (B) Coronary codominance. (C) Left coronary artery dominance. The functional Duke Jeopardy score system categorizes the coronary artery tree into six segments for patients with right coronary artery (RCA) dominance and coronary codominance: the left anterior descending artery (LAD), the largest diagonal branch (D), the largest septal perforating branch (Sept), the left circumflex artery (LCX), the largest obtuse marginal branch (OM), and the posterior descending artery of RCA (R-PDA). A CT-FFR value ≤0.80 in any segment results in a score of 2, otherwise it is scored as 0. In cases of left coronary artery dominance, the scoring system omits R-PDA and reallocates its points to the posterior descending artery of LCX (L-PDA). Each segment can score 2 points, allowing for a maximum score of 12. CT-FFR, coronary computed tomography angiography-derived fractional flow reserve; fDJS_CTA_, CT-FFR based functional Duke Jeopardy score.

The Coronary Artery Disease-Reporting and Data System (CAD-RADS) [62] is the cornerstone of standardized CCTA reporting and has evolved from its original anatomical classification to the updated 2022 CAD-RADS 2.0 version [63]. This version integrates stenosis, plaque burden, and functional assessment through modifier “I” when CT-FFR or myocardial CT perfusion data is available. Table 3 summarizes the interpretation of modifier “I” for CT-FFR in CAD-RADS 2.0. Several CT-FFR-based scoring systems have emerged from this integration of functional parameters (Table 4, Ref. [64, 65, 66, 67, 68]), including the CT-FFR-based functional SYNTAX score, CT-FFR-based functional CAD-RADS (Fig. 2), and the CT-FFR based functional Duke Jeopardy Score (Fig. 3). Each of these systems is designed to improve specific aspects of risk stratification and treatment planning [64, 65, 66, 67, 68]. A significant proportion (22.9%) of patients initially categorized as high/intermediate risk were down-classified to low-risk by the CT-FFR-based functional SYNTAX score (FSS_CTA_). Furthermore, management plans were modified in 30% of cases following functional CAD-RADS assessment, diverging from the recommendations based on anatomical CAD-RADS. CT-FFR-based functional scoring systems have demonstrated substantial clinical impact through improved risk stratification and treatment decision-making, thus offering a more precise and personalized approach to CAD management.

**Table 3.** **The interpretation of modifier “I” for CT-FFR in CAD-RADS 2.0**.

CT-FFR	Interpretation and Considerations
Abnormal (I+)	CAD-RADS 3 or 4/I+
≤0.75	Anatomical stenosis matches with hemodynamic significance, and revascularization is recommended.
Borderline (I±)	CAD-RADS 3 or 4/I±
0.76 to 0.80	Indications for invasive angiography include relevant symptoms, lesion location, and a hemodynamically significant ΔCT-FFR (>0.12), quantified as the proximal-to-distal CT-FFR difference measured within 1–2 cm of the stenosis.
Normal (I–)	CAD-RADS 3 or 4/I–
>0.80	Anatomic stenosis mismatches with ischemic lesions, or hemodynamic significance present without stenosis. Deferral of invasive coronary angiography and the optimization of medical therapy are recommended.

CT-FFR is clinically indicated for hemodynamic assessment of 50–90% coronary stenoses, specifically CAD-RADS 3 and 4A lesions. For CAD-RADS 2 with proximal ≥40% stenosis, CT-FFR may be considered to assess the hemodynamic significance even with high-risk plaque features. CT-FFR, computed tomography-derived fractional flow reserve; CAD-RADS, Coronary Artery Disease-Reporting and Data System.

**Table 4.** **CT-FFR-based functional scoring systems**.

Scoring system	Basic principle	Key findings
Functional SYNTAX score (FSS) [64]	Only lesions that are functionally significant (FFR ≤0.80) are included in the FSS calculation.	Non-invasive FSS achieved 30% reclassification from high/intermediate-risk to low-risk categories, compared 23% with invasive FSS.
	Lesions with FFR >0.80 are not counted in the score, even if they appear to be anatomically significant.	Technically feasible and inter-modality concordance (Kappa = 0.32) were validated.
SYNTAX score III [65]	**Step 1**. The non-invasive functional SYNTAX score is derived by excluding non-flow-limiting lesions (CT-FFR >0.80) from the anatomic SYNTAX assessment.	CT-FFR integration modified CCTA-guided treatment decisions for 7% patients, concurrently adjusting revascularization strategies in 12.1% of vessels.
	**Step 2**. SYNTAX Score III integrates this functional adjusted score with the patient’s clinical profile, thereby integrating coronary anatomy complexity, functional significance, and clinical factors.	The addition of CT-FFR to conventional angiography altered treatment decisions in 6.6% of patients, and revised management strategies in 18.3% of cases.
		CT-FFR integration prompted SYNTAX score down-reclassification in 15.5% when assessed by CCTA, compared to 14% re-stratification based on conventional angiography.
		CT-FFR reassessment reduced the prevalence of significant triple-vessel CAD identified by CCTA from 92.3% to 78.8%.
CT-FFR-based functional SYNTAX score (FSS_CTA_) [66]	Only lesions with CT-FFR <0.80 are included in the final FSS_CTA_ calculation.	FSS_CTA_ facilitated risk reclassification in 22.9% of patients, redistributing them from high/intermediate to low-risk groups.
	The score combines both the anatomical data from CCTA and functional data from CT-FFR.	Patients reclassified to the low-risk group showed a lower incidence of MACE (3.2% vs. 34.3%, *p * < 0.001).
CT-FFR-based functional CAD-RADS [67]	Integrates CT-FFR results with anatomical CAD-RADS.	Functional CAD-RADS outperformed anatomical CAD-RADS in therapeutic guidance (AUC: 0.828 vs. 0.681, *p * < 0.001), prompting management modifications in 30% of patients.
	Reclassification rules:	This integrated approach independently predicted 1-year adverse outcomes.
	∙ CAD-RADS 2/3 lesions are elevated to functional CAD-RADS 3/4 when demonstrating hemodynamic significance (CT-FFR ≤0.80) in any vessel.	
	∙ CAD-RADS 4 lesions are revised to functional CAD-RADS 3 if CT-FFR >0.80 in all vessels.	
CT-FFR-based functional Duke Jeopardy Score (fDJS_CTA_) [68]	Uses CT-FFR values to assess the severity of CAD.	fDJS_CTA_ proved to be the strongest predictor for MACE, with a hazard ratio of 7.08 over three years of follow-up.
	Assigns 2 points for segments with CT-FFR ≤0.80, and 0 points if CT-FFR is >0.80.	The predictive accuracy of fDJS_CTA_ remained strong over time, with time-dependent AUC values of 0.89 at one year, 0.83 at two years, and 0.82 at three years.
	The maximum score is 12 points.	fDJS_CTA_ improved risk classification in 19.7% of patients compared to traditional CT-FFR methods.

CT-FFR, computed tomography-derived fractional flow reserve; CCTA, coronary computed tomography angiography; FFR, fractional flow reserve; FSS, functional SYNTAX score; FSS_CTA_, CT-FFR-based functional SYNTAX score; CAD-RADS, Coronary Artery Disease-Reporting and Data System; fDJS_CTA_, CT-FFR based functional Duke Jeopardy Score; CAD, coronary artery disease; MACE, major adverse cardiovascular events; AUC, area under the curve.

## 6. Prognostic Evaluation

Long-term follow-up studies, particularly the landmark ADVANCE trial (Assessing Diagnostic Value of Non-invasive FFR_CT_ in Coronary Care) [69], have established the prognostic significance of CT-FFR. This 3-year prospective study with 900 stable angina participants demonstrated a 3.2-fold higher risk of all-cause mortality and spontaneous myocardial infarction with CT-FFR values ≤0.80 versus >0.80. The low-CT-FFR cohort exhibited 8.8-fold elevated cardiovascular mortality and spontaneous myocardial infarction risk, confirming CT-FFR’s prognostic value for CAD risk stratification. Other studies have also shown that CT-FFR can improve the prediction of cardiovascular events [70, 71, 72, 73, 74, 75]. Current evidence indicates that successful implementation of artificial intelligence-based, fully-automated on-site CT-FFR in emergency departments can quickly and reliably identify patients at higher risk of MACE, enabling timely interventions and potentially improving outcomes [8]. Patients with positive CT-FFR experienced a significantly higher incidence of MACE (44.3%) compared to those with negative CT-FFR, who had a MACE incidence of only 4.2%. Research has shown that certain metrics, such as remodeling index ≥1.10 and CT-FFR value ≤0.85, are independent predictors of plaque progression [73]. Moreover, a decreased lesion-specific CT-FFR and an increased fat attenuation index were associated with a higher risk of MACE [74]. Artificial intelligence-driven integration of quantitative plaque characterization and hemodynamic assessment was found to significantly improve the prediction of acute coronary syndrome culprit lesions compared to conventional CCTA metrics (AUC: 0.84 vs. 0.78, *p* < 0.001) [76]. Both plaque progression and CT-FFR provided incremental predictive value for MACE, surpassing traditional markers such as CACS and the presence of high-risk plaque.

In patients with CAD, the addition of CT-FFR and/or resting static myocardial CT perfusion can help guide subsequent treatment and reduce the incidence of MACE [77, 78]. The three CT-FFR-based functional scoring systems (CT-FFR-based functional SYNTAX score, functional CAD-RADS, and functional Duke Jeopardy Score) show superior predictive value for CAD prognosis compared to conventional methods [66, 67, 68]. These methodologies enhance prognostic capabilities, refine risk assessment, and enable more tailored risk stratification strategies, allowing clinicians to more accurately evaluate plaque progression, improve the predictive accuracy of CCTA, and establish a foundation for timely clinical interventions and treatments.

## 7. Standardization and Promotion of CT-FFR

### 7.1 Development of Expert Consensus and a Group Standard

**Fig. 4.**
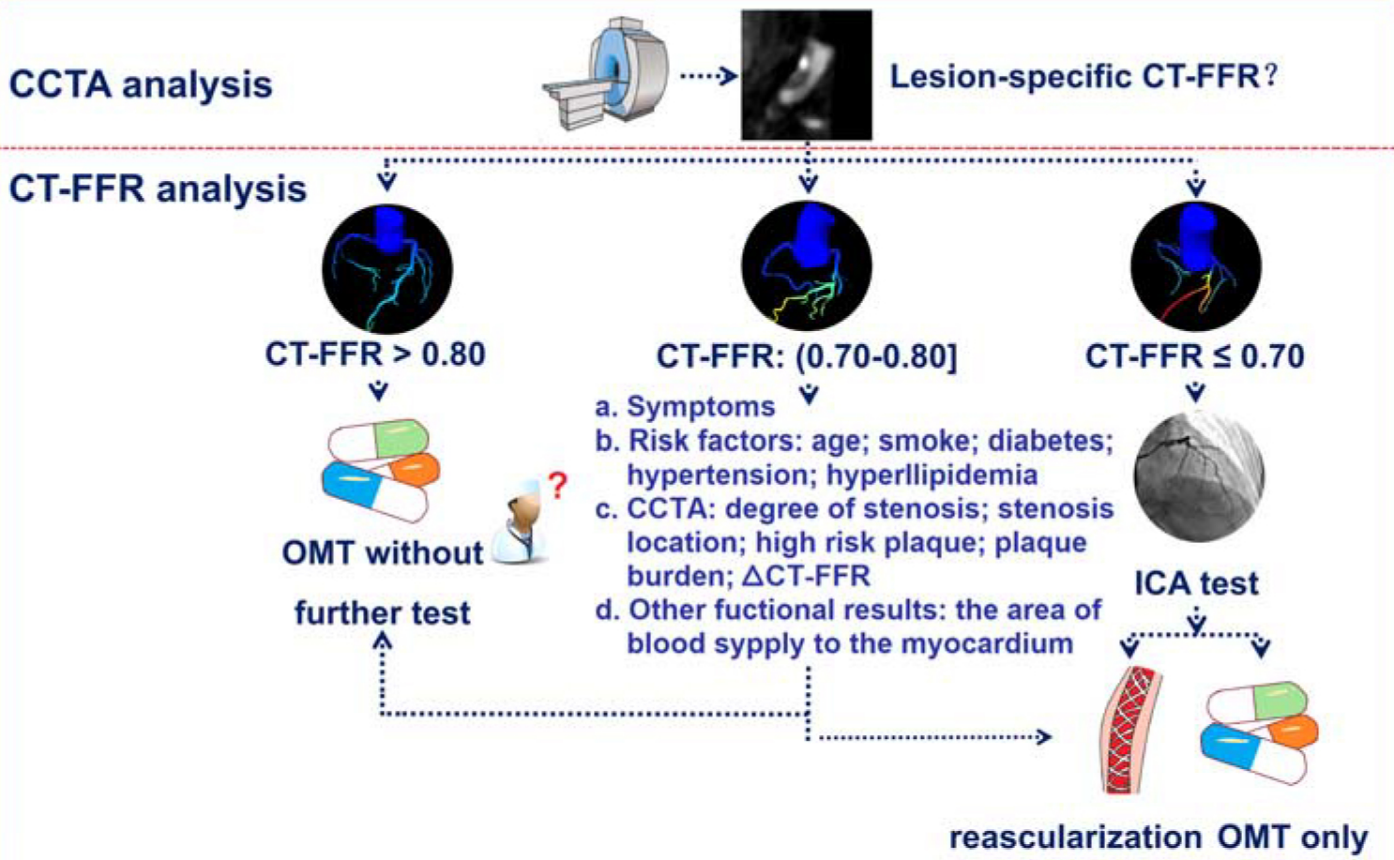
**Flowchart of CT-FFR applications and interpretation**. CT-FFR, coronary computed tomography angiography-derived fractional flow reserve; CCTA, coronary computed tomography angiography; OMT, optimal medical therapy; ICA, invasive coronary angiography. (Reprinted from Zhang *et al*. [51] with permission from Wolters Kluwer Health).

The introduction of CT-FFR underscores the significant progress made in the non-invasive evaluation of CAD, garnering widespread interest in the world. Several expert recommendations and consensus documents have been published [51, 79, 80, 81]. In particular, the “Coronary computed tomography angiography-derived fractional flow reserve: an expert consensus document of the Chinese Society of Radiology” provides a roadmap for incorporating CT-FFR into CAD diagnostics (Fig. 4, Ref. [51]).

### 7.2 Accumulation of Clinical Evidence for CT-FFR

Multiple ongoing multicenter trials are currently investigating the clinical value and cost-benefit profile of CCTA/CT-FFR integrated implementation. Notable amongst these trials are ADVANCE [69], PLATFORM (Prospective Longitudinal Trial of FFR_CT_: Outcome and Resource Impacts Study) [82], FORECAST (Fractional Flow Reserve Derived from Computed Tomography Angiography in the Assessment and Management of Stable Chest Pain) [83], and PRECISE (Prospective Randomized Trial of the Optimal Evaluation of Cardiac Symptoms and Revascularization) [84]. The aim of such trials is to assess whether a combined CCTA/CT-FFR approach can optimize clinical decision pathways, and enhance patient outcomes across diverse healthcare settings while remaining cost-effective. Initial findings from real-world studies have yielded mixed results. A multicenter study conducted in the United Kingdom reported limitations in the ability of CT-FFR to predict significant CAD, while noting increased costs due to higher rates of invasive procedures [85]. Selective CT-FFR with CCTA was shown in the FORECAST study to reduce ICA referrals among stable angina cases, though cost and clinical outcomes remained statistically indistinguishable from conventional management [83]. These findings were corroborated by the Chinese TARGET trial (Effect of On-Site CT-Derived Fractional Flow Reserve on the Management of Decision Making for Patients With Stable Chest Pain) [12]. Therefore, existing evidence on CT-FFR’s cost-effectiveness exhibits significant heterogeneity across clinical settings. Beyond reducing unnecessary ICA, a comprehensive economic evaluation should also include the broader value proposition of CT-FFR, including patient benefits from a non-invasive “one-stop-shop” approach, potential long-term improvements in outcome due to optimized revascularization decisions, and overall gains in system efficiency. Further investigation is required to comprehensively evaluate the long-term economic outcomes and implementation feasibility, particularly in the context of diverse healthcare systems such as those found in China. Two prospective multicenter studies are currently underway. The China CT-FFR study 2 (Chinese Multicenter Assessment of CT-derived Fractional Flow Reserve 2) enrolled more than 10,000 participants with non-obstructive CAD across 29 centers in China, with the aim of evaluating the predictive value of CT-FFR for MACE in non-obstructive CAD patients [86]. One-year follow up results from the China CT-FFR study 3 [87] have shown that adding CT-FFR to CCTA could reduce the 90-day ICA rate by 19.4% in a Chinese real-word setting. Assessment of longer term prognostic value requires further study. These comprehensive clinical trials are anticipated to enhance the integration of CT-FFR in clinical practice, ultimately improving patient outcomes.

## 8. Limitations of CT-FFR

Despite established utility in diagnosing CAD and informing treatment strategies, CT-FFR is subject to several limitations: (1) Different CT-FFR methodologies have their own strengths and weaknesses. Further comparative studies are needed to evaluate their respective performance and optimize their clinical applications. (2) Diagnostic accuracy is significantly compromised by the reliance on high image quality and by specific high-risk scenarios. For example, severe calcification (inducing blooming artifacts), motion/noise artifacts, and suboptimal contrast can directly undermine reliability. Unreliable results are particularly prevalent in cases of arrhythmias, uncontrolled heart rates, or complex bifurcations. (3) The clinical indications for CT-FFR require further study. Currently, there is insufficient evidence regarding its applicability in specific patient populations, such as post-percutaneous coronary intervention (PCI) patients and those with a myocardial bridge. (4) The long-term prognostic value of CT-FFR in clinical practice needs to be validated in clinical trials. (5) The cost-effectiveness of implementing CT-FFR warrants more comprehensive investigation. (6) CT-FFR should not be considered as the sole indicator for CAD evaluation. Integration with other parameters, particularly plaque characteristics, is essential for improving the diagnostic and prognostic performance.

## 9. Conclusions

The integration of CT-FFR with CCTA has become a powerful diagnostic approach for comprehensive CAD assessment. Recently developed on-site CT-FFR strategies, particularly artificial intelligence-based fully automated on-site CT-FFR, have shown promising diagnostic accuracy, thereby reducing unnecessary ICA rates and improving the prognostic capability. These advances enable a more personalized approach to managing suspected CAD, ultimately improving patient care through precision medicine.

## Abbreviations

AUC, area under the curve; CABG, coronary artery bypass grafting; CACS, coronary artery calcium score; CAD, coronary artery disease; CAD-RADS, Coronary Artery Disease-Reporting and Data System; CCTA, coronary computed tomography angiography; CFD, computational fluid dynamics; CT-FFR, coronary computed tomography angiography-derived fractional flow reserve; DL, deep learning; FSS_CTA_, CT-FFR-based functional SYNTAX score; fDJS_CTA_, CT-FFR based functional Duke Jeopardy score; FFR, fractional flow reserve; ICA, invasive coronary angiography; MACE, major adverse cardiovascular events; MB, myocardial bridge; ML, machine learning; PCCT, photon-counting detector computed tomography; PCI, percutaneous coronary intervention; TAVR, transcatheter aortic valve replacement.

## Supplementary Material

Supplementary material associated with this article can be found, in the online version, at https://doi.org/10.31083/RCM39717.
