# Supplementary material for: Coronary Computed Tomography Angiography-Derived Fractional Flow Reserve: A Comprehensive Review
Source: Rev Cardiovasc Med. 2025 Sep 26;26(9):39717. doi: 10.31083/RCM39717 (PMC12516765; doi:10.31083/RCM39717)
Supplement: Supplementary file 1 [file 2153-8174-26-9-39717-s1.docx]

**Supplementary Materials**

**Supplementary Table 1** Per-vessel and/or per-patient diagnostic performance of HeartFlow^®^ CT-FFR (Redwood City, California, USA) compared with CCTA

| **Study** | **Design** | **Reference** | **N** | **Acc. (%)** | | **Sen. (%)** | | **Spec. (%)** | | **PPV (%)** | | **NPV (%)** | | **AUC (%)** | | |
| --- | --- | --- | --- | --- | --- | --- | --- | --- | --- | --- | --- | --- | --- | --- | --- | --- |
|  |  |  |  | **CT-FFR** | **CCTA** | **CT-FFR** | **CCTA** | **CT-FFR** | **CCTA** | **CT-FFR** | **CCTA** | **CT-FFR** | **CCTA** | **CT-FFR** | **CCTA** | |
| DISCOVER-FLOW [1] | Prospective,  multicenter | **On a per-vessel basis** | | | | | | | | | | | | | |  |
|  |  | FFR ≤ 0.80 | 159 | 84.3 | 58.5 | 87.9 | 91.4 | 82.2 | 39.6 | 73.9 | 46.5 | 92.2 | 88.9 | 90 | 75 | |
|  |  | **On a per-patient basis** | | | | | | | | | | | | | |  |
|  |  | FFR ≤ 0.80 | 103 | 87.4 | 61.2 | 92.6 | 94.4 | 81.6 | 24.5 | 84.7 | 58 | 90.9 | 80 | 92 | 70 | |
| DeFACTO [2] | Prospective,  multicenter | **On a per-patient basis** | | | | | | | | | | | | | |  |
|  |  | FFR ≤ 0.80 | 252 | 73 | 64 | 90 | 84 | 54 | 42 | 67 | 61 | 84 | 72 | 81 | 68 | |
| NXT [3] | Prospective,  multicenter | **On a per-vessel basis** | | | | | | | | | | | | | |  |
|  |  | FFR ≤ 0.80 | 484 | 86 | 65 | 84 | 83 | 86 | 60 | 61 | 33 | 95 | 92 | 93 | 79 | |
|  |  | **On a per-patient basis** | | | | | | | | | | | | | |  |
|  |  | FFR ≤ 0.80 | 254 | 81 | 53 | 86 | 94 | 79 | 34 | 65 | 40 | 93 | 92 | 90 | 81 | |
| Driessen et al [4] | Prospective,  single-center | **On a per-vessel basis** | | | | | | | | | | | | | |  |
|  |  | FFR ≤ 0.80 | 505 | 87 | 79 | 90 | 68 | 86 | 83 | 65 | 57 | 96 | 86 | 94 | 83 | |
|  |  | **On a per-patient basis** | | | | | | | | | | | | | |  |
|  |  | FFR ≤ 0.80 | 157 | 78 | 76 | 96 | 87 | 63 | 67 | 68 | 69 | 95 | 87 | 78 | 76 | |
| Pontone et al [5] | Prospective,  single-center | **On a per-vessel basis** | | | | | | | | | | | | | |  |
|  |  | FFR ≤ 0.8 or ICA stenosis > 80% or total occlusion | 441 | 92 | 82 | 88 | 99 | 94 | 76 | 84 | 61 | 95 | 100 | 93 | 89 | |
|  |  | **On a per-patient basis** | | | | | | | | | | | | | |  |
|  |  | FFR ≤ 0.8 or ICA stenosis > 80% or total occlusion | 147 | 87 | 73 | 90 | 95 | 85 | 54 | 83 | 63 | 92 | 94 | 94 | 90 | |

CT-FFR, coronary computed tomography angiography-derived fractional flow reserve; CCTA, coronary computed tomography angiography; ICA, invasive coronary angiography; Acc., accuracy; Sen., sensitivity; Spec., specificity; PPV, positive predictive value; NPV, negative predictive value; AUC, area under the curve**Supplementary Table 2** Per-vessel and/or per-patient diagnostic performance of cFFR (Siemens Healthineers, Erlangen, Germany) compared with CCTA

| **cFFR version** | **Design** | **Reference** | **N** | **Acc. (%)** | | **Sen. (%)** | | **Spec. (%)** | | **PPV (%)** | | **NPV (%)** | | **AUC (%)** | |
| --- | --- | --- | --- | --- | --- | --- | --- | --- | --- | --- | --- | --- | --- | --- | --- |
|  |  |  |  | **CT-FFR** | **CCTA** | **CT-FFR** | **CCTA** | **CT-FFR** | **CCTA** | **CT-FFR** | **CCTA** | **CT-FFR** | **CCTA** | **CT-FFR** | **CCTA** |
| cFFR version 1.4 [6] | Retrospective,  single-center | **On a per-vessel basis** | | | | | | | | | | | | | |
|  |  | FFR ≤ 0.80 | 189 | 75 | 56 | 88 | 81 | 65 | 38 | 65 | 49 | 88 | 73 | 83 | 64 |
| cFFR version 2.1 [7] | Prospective and retrospective,  multicenter | **On a per-vessel basis** | | | | | | | | | | | | | |
|  |  | FFR ≤ 0.80 | 525 | 78 | 58 | 81 | 88 | 76 | 38 | 70 | 49 | 85 | 83 | 84 | 69 |
|  |  | **On a per-patient basis** | | | | | | | | | | | | | |
|  |  | FFR ≤ 0.80 or ICA stenosis > 90% | 303 | 85 | 71 | 89 | 99 | 76 | 11 | 89 | 71 | 77 | 77 | - | - |
| cFFR version 3.0 [8] | Prospective,  single-center | **On a per-vessel basis** | | | | | | | | | | | | | |
|  |  | FFR ≤ 0.8 | 127 | 72 | 64 | 83 | 74 | 63 | 57 | 63 | 57 | 83 | 74 | 79 | 67 |
|  |  | **On a per-patient basis** | | | | | | | | | | | | | |
|  |  | FFR ≤ 0.80 | 72 | 78 | 65 | 70 | 78 | 88 | 50 | 88 | 66 | 70 | 64 | 85 | 66 |
| cFFR version 3.0 [9] | Retrospective,  multicenter | **On a per-vessel basis** | | | | | | | | | | | | | |
|  |  | FFR ≤ 0.8 | 183 | 90 | 55 | 84 | 95 | 94 | 28 | 90 | 47 | 90 | 88 | 95 | 61 |
|  |  | **On a per-patient basis** | | | | | | | | | | | | | |
|  |  | FFR ≤ 0.8 | 136 | 89 | 63 | 86 | 95 | 92 | 33 | 90 | 56 | 88 | 89 | 95 | 64 |
| cFFR version 3.2 [10] | Prospective,  multicenter | **On a per-vessel basis** | | | | | | | | | | | | | |
|  |  | FFR ≤ 0.80 or ICA stenosis > 90% | 271 | 90 | 79 | 86 | 95 | 91 | 74 | 75 | 54 | 96 | 98 | 84 | - |
|  |  | **On a per-patient basis** | | | | | | | | | | | | | |
|  |  | FFR ≤ 0.80 or ICA stenosis > 90% | 105 | 83 | 72 | 90 | 94 | 77 | 54 | 77 | 64 | 90 | 91 | - | - |

CT-FFR, coronary computed tomography angiography-derived fractional flow reserve; CCTA, coronary computed tomography angiography; ICA, invasive coronary angiography; Acc., accuracy; Sen., sensitivity; Spec., specificity; PPV, positive predictive value; NPV, negative predictive value; AUC, area under the curve

**Supplementary Table 3** Per-vessel and/or per-patient diagnostic performance of China's domestic CT-FFR compared with CCTA

| **Study** | **Design** | **Reference** | **N** | **Acc. (%)** | | **Sen. (%)** | | **Spec. (%)** | | **PPV (%)** | | **NPV (%)** | | **AUC (%)** | |
| --- | --- | --- | --- | --- | --- | --- | --- | --- | --- | --- | --- | --- | --- | --- | --- |
|  |  |  |  | **CT-FFR** | **CCTA** | **CT-FFR** | **CCTA** | **CT-FFR** | **CCTA** | **CT-FFR** | **CCTA** | **CT-FFR** | **CCTA** | **CT-FFR** | **CCTA** |
| Tang et al [11] | Retrospective,  multicenter | **On a per-vessel basis** | | | | | | | | | | | | | |
|  |  | FFR ≤ 0.80 | 422 | 91 | 55 | 89 | 92 | 91 | 34 | 86 | 45 | 94 | 88 | 92 | 75 |
|  |  | **On a per-patient basis** | | | | | | | | | | | | | |
|  |  | FFR ≤ 0.80 | 338 | 90 | 56 | 89 | 92 | 91 | 30 | 88 | 49 | 92 | 83 | 92 | 76 |
| Jiang et al [12] | Retrospective,  multicenter | **On a per-vessel basis** | | | | | | | | | | | | | |
|  |  | FFR ≤ 0.80 | 190 | 91.05 | 40 | 92.73 | 96.36 | 90.37 | 17.04 | 79.69 | 32.12 | 96.83 | 92 | 92.7 | 76.4 |
|  |  | **On a per-patient basis** | | | | | | | | | | | | | |
|  |  | FFR ≤ 0.80 | 146 | 91.78 | 45.21 | 92.31 | 94.23 | 91.49 | 18.09 | 85.71 | 38.89 | 95.56 | 85 | 93.5 | 76.1 |
| Wang et al [13] | Prospective,  single-center | **On a per-vessel basis** | | | | | | | | | | | | | |
|  |  | FFR ≤ 0.80 | 71 | 88.73 | - | 97.56 | - | 76.67 | - | 85.11 | - | 95.83 | - | 93.3 | 66.2 |
|  |  | **On a per-patient basis** | | | | | | | | | | | | | |
|  |  | FFR ≤ 0.80 | 63 | 87.3 | - | 97.14 | - | 75 | - | 82.93 | - | 95.45 | - | 92.8 | 66.4 |
| Guo et al [14] | Retrospective,  multicenter | **On a per-vessel basis** | | | | | | | | | | | | | |
|  |  | FFR ≤ 0.80 | 600 | 82 | 48 | 80 | 90 | 83 | 22 | 74 | 42 | 87 | 77 | 82 | 56 |
|  |  | **On a per-patient basis** | | | | | | | | | | | | | |
|  |  | FFR ≤ 0.80 | 463 | 82 | 50 | 84 | 92 | 81 | 18 | 77 | 46 | 86 | 73 | 82 | 55 |
| Hou et al [15] | Prospective,  single-center | **On a per-patient basis** | | | | | | | | | | | | | |
|  |  | FFR ≤ 0.80 | 73 | 87.6 | 54.7 | 87 | 52.2 | 88 | 50 | 76.9 | 35.2 | 93.6 | 71.7 | 83.9 | 51.1 |
| Ding et al [16] | Prospective,  multicenter | **On a per-vessel basis** | | | | | | | | | | | | | |
|  |  | FFR ≤ 0.80 | 324 | 94 | 71 | 95 | 82 | 92 | 62 | 91 | 64 | 96 | 80 | 97 | 60 |
|  |  | **On a per-patient basis** | | | | | | | | | | | | | |
|  |  | FFR ≤ 0.80 | 303 | 94 | 79 | 96 | 94 | 92 | 65 | 91 | 70 | 96 | 92 | 97 | 64 |
| Gao et al [17] | Prospective,  multicenter | **On a per-vessel basis** | | | | | | | | | | | | | |
|  |  | FFR < 0.80 | 366 | 88.8 | 60.4 | 89.9 | 89.3 | 87.8 | 35.5 | 87.3 | 54.3 | 91.1 | 79.5 | 89 | 66 |
|  |  | **On a per-patient basis** | | | | | | | | | | | | | |
|  |  | FFR < 0.80 | 317 | 90.5 | 63.4 | 90.7 | 88.9 | 90.4 | 37.2 | 90.7 | 59.3 | 90.4 | 76.3 | 92 | 64 |
| Jiang et al [18] | Retrospective,  single-center | **On a per-vessel basis** | | | | | | | | | | | | | |
|  |  | FFR ≤ 0.80 | 78 | 89.7 | 32 | 90.5 | 100 | 89.5 | 7 | 76 | - | 96.2 | - | 92.5 | 85.3 |
|  |  | **On a per-patient basis** | | | | | | | | | | | | | |
|  |  | FFR ≤ 0.80 | 54 | 90.7 | 38.9 | 89.5 | 100 | 91.4 | 5.71 | 85 | 36.5 | 94.1 | 100 | 94.5 | 87 |
| Li et al [19] | Prospective,  multicenter | **On a per-vessel basis** | | | | | | | | | | | | | |
|  |  | FFR ≤ 0.80 | 404 | 90.6 | 54.3 | 90.9 | 86.6 | 90.4 | 34.7 | 85.3 | 44.6 | 94.2 | 81 | 93 | 77 |
|  |  | **On a per-patient basis** | | | | | | | | | | | | | |
|  |  | FFR ≤ 0.80 | 339 | 90.3 | 53.4 | 91.4 | 87.1 | 89.5 | 29.4 | 85.9 | 46.7 | 93.7 | 76.3 | 92 | 76 |
| Wen et al [20] | Retrospective,  single-center | **On a per-vessel basis** | | | | | | | | | | | | | |
|  |  | FFR ≤ 0.80 | 89 | 87.6 | 44.9 | 86.1 | 80.6 | 88.7 | 20.8 | 83.8 | 40.8 | 90.4 | 61.1 | 87.4 | 50.7 |

CT-FFR, coronary computed tomography angiography-derived fractional flow reserve; CCTA, coronary computed tomography angiography; Acc., accuracy; Sen., sensitivity; Spec., specificity; PPV, positive predictive value; NPV, negative predictive value; AUC, area under the curve


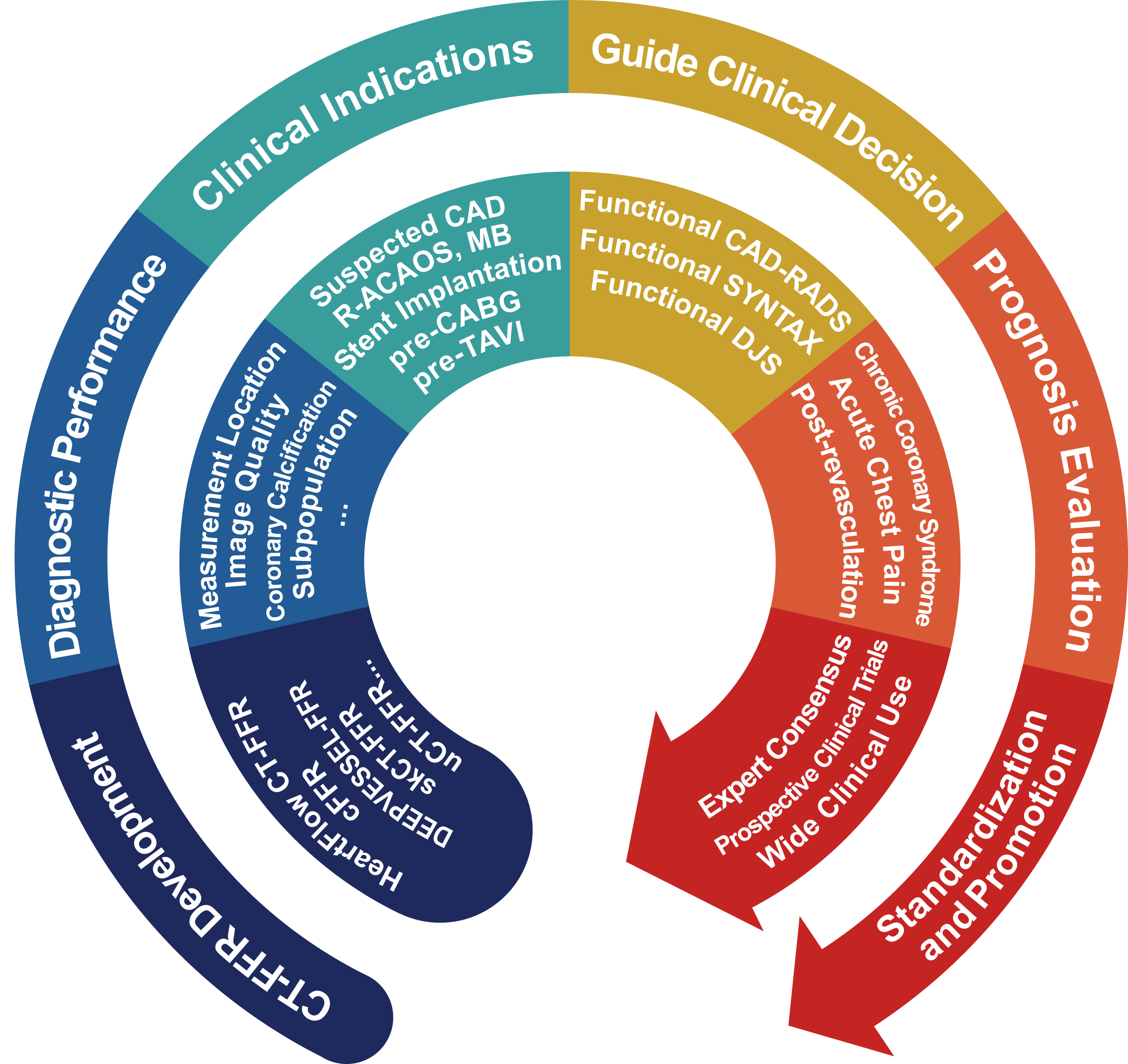


**Supplementary Fig. 1:** **An overview of CT-FFR development and research.**

CT-FFR, coronary computed tomography angiography-derived fractional flow reserve; R-ACAOS, anomalous origin of the right coronary artery from the left coronary sinus; MB, myocardial bridge; CABG, coronary artery bypass grafting; TAVI, transcatheter aortic valve implantation; CAD-RADS, Coronary Artery Disease-Reporting and Data System; DJS, Duke Jeopardy Score

**References**

[1] Koo BK, Erglis A, Doh JH, Daniels DV, Jegere S, Kim HS, et al. Diagnosis of ischemia-causing coronary stenoses by noninvasive fractional flow reserve computed from coronary computed tomographic angiograms. Results from the prospective multicenter DISCOVER-FLOW (Diagnosis of Ischemia-Causing Stenoses Obtained Via Noninvasive Fractional Flow Reserve) study. J Am Coll Cardiol. 2011; 58: 1989-1997. <https://doi.org/10.1016/j.jacc.2011.06.066>

[2] Min JK, Leipsic J, Pencina MJ, Berman DS, Koo BK, van Mieghem C, et al. Diagnostic accuracy of fractional flow reserve from anatomic CT angiography. Jama. 2012; 308: 1237-1245. <https://doi.org/10.1001/2012.jama.11274>

[3] Nørgaard BL, Leipsic J, Gaur S, Seneviratne S, Ko BS, Ito H, et al. Diagnostic performance of noninvasive fractional flow reserve derived from coronary computed tomography angiography in suspected coronary artery disease: the NXT trial (Analysis of Coronary Blood Flow Using CT Angiography: Next Steps). J Am Coll Cardiol. 2014; 63: 1145-1155. <https://doi.org/10.1016/j.jacc.2013.11.043>

[4] Driessen RS, Danad I, Stuijfzand WJ, Raijmakers PG, Schumacher SP, van Diemen PA, et al. Comparison of Coronary Computed Tomography Angiography, Fractional Flow Reserve, and Perfusion Imaging for Ischemia Diagnosis. J Am Coll Cardiol. 2019; 73: 161-173. <https://doi.org/10.1016/j.jacc.2018.10.056>

[5] Pontone G, Baggiano A, Andreini D, Guaricci AI, Guglielmo M, Muscogiuri G, et al. Stress Computed Tomography Perfusion Versus Fractional Flow Reserve CT Derived in Suspected Coronary Artery Disease: The PERFECTION Study. JACC Cardiovasc Imaging. 2019; 12: 1487-1497. <https://doi.org/10.1016/j.jcmg.2018.08.023>

[6] Coenen A, Lubbers MM, Kurata A, Kono A, Dedic A, Chelu RG, et al. Fractional flow reserve computed from noninvasive CT angiography data: diagnostic performance of an on-site clinician-operated computational fluid dynamics algorithm. Radiology. 2015; 274: 674-683. <https://doi.org/10.1148/radiol.14140992>

[7] Coenen A, Kim YH, Kruk M, Tesche C, De Geer J, Kurata A, et al. Diagnostic Accuracy of a Machine-Learning Approach to Coronary Computed Tomographic Angiography-Based Fractional Flow Reserve: Result From the MACHINE Consortium. Circ Cardiovasc Imaging. 2018; 11: e007217. <https://doi.org/10.1161/circimaging.117.007217>

[8] Li Y, Yu M, Dai X, Lu Z, Shen C, Wang Y, et al. Detection of Hemodynamically Significant Coronary Stenosis: CT Myocardial Perfusion versus Machine Learning CT Fractional Flow Reserve. Radiology. 2019; 293: 305-314. <https://doi.org/10.1148/radiol.2019190098>

[9] Tang CX, Wang YN, Zhou F, Schoepf UJ, Assen MV, Stroud RE, et al. Diagnostic performance of fractional flow reserve derived from coronary CT angiography for detection of lesion-specific ischemia: A multi-center study and meta-analysis. Eur J Radiol. 2019; 116: 90-97. <https://doi.org/10.1016/j.ejrad.2019.04.011>

[10] Soschynski M, Storelli R, Birkemeyer C, Hagar MT, Faby S, Schwemmer C, et al. CT Myocardial Perfusion and CT-FFR versus Invasive FFR for Hemodynamic Relevance of Coronary Artery Disease. Radiology. 2024; 312: e233234. <https://doi.org/10.1148/radiol.233234>

[11] Tang CX, Liu CY, Lu MJ, Schoepf UJ, Tesche C, Bayer RR, 2nd, et al. CT FFR for ischemia-specific CAD with a new computational fluid dynamics algorithm: a Chinese multicenter study. JACC Cardiovasc Imaging. 2020; 13: 980-990. <https://doi.org/10.1016/j.jcmg.2019.06.018>

[12] Jiang J, Du C, Hu Y, Yuan H, Wang J, Pan Y, et al. Diagnostic performance of computational fluid dynamics (CFD)-based fractional flow reserve (FFR) derived from coronary computed tomographic angiography (CCTA) for assessing functional severity of coronary lesions. Quant Imaging Med Surg. 2023; 13: 1672-1685. <https://doi.org/10.21037/qims-22-521>

[13] Wang ZQ, Zhou YJ, Zhao YX, Shi DM, Liu YY, Liu W, et al. Diagnostic accuracy of a deep learning approach to calculate FFR from coronary CT angiography. J Geriatr Cardiol. 2019; 16: 42-48. <https://doi.org/10.11909/j.issn.1671-5411.2019.01.010>

[14] Guo BJ, Jiang MC, Guo X, Tang CX, Zhong J, Lu MJ, et al. Diagnostic and prognostic performance of artificial intelligence-based fully-automated on-site CT-FFR in patients with CAD. Sci Bull (Beijing). 2024; 69: 1472-1485. <https://doi.org/10.1016/j.scib.2024.03.053>

[15] Hou C, Lu Y, Ma Y, Li Q, Liu C, Lu M, et al. Investigation of the predictive value of a novel algorithm based on coronary CT angiography regarding fractional flow reserve and revascularization in patients with stable coronary artery disease. Heart Vessels. 2024; 39: 195-205. <https://doi.org/10.1007/s00380-023-02324-y>

[16] Ding Y, Li Q, Zhang Y, Tang Y, Zhang H, Yang Q, et al. Diagnostic accuracy of noninvasive fractional flow reserve derived from computed tomography angiography in ischemia-specific coronary artery stenosis and indeterminate lesions: results from a multicenter study in China. Front Cardiovasc Med. 2023; 10: 1236405. <https://doi.org/10.3389/fcvm.2023.1236405>

[17] Gao Y, Zhao N, Song L, Hu H, Jiang T, Chen W, et al. Diagnostic Performance of CT FFR With a New Parameter Optimized Computational Fluid Dynamics Algorithm From the CT-FFR-CHINA Trial: Characteristic Analysis of Gray Zone Lesions and Misdiagnosed Lesions. Front Cardiovasc Med. 2022; 9: 819460. <https://doi.org/10.3389/fcvm.2022.819460>

[18] Jiang W, Pan Y, Hu Y, Leng X, Jiang J, Feng L, et al. Diagnostic accuracy of coronary computed tomography angiography-derived fractional flow reserve. Biomed Eng Online. 2021; 20: 77. <https://doi.org/10.1186/s12938-021-00914-3>

[19] Li C, Hu Y, Jiang J, Dong L, Sun Y, Tang L, et al. Diagnostic Performance of Fractional Flow Reserve Derived From Coronary CT Angiography: The ACCURATE-CT Study. JACC Cardiovasc Interv. 2024; 17: 1980-1992. <https://doi.org/10.1016/j.jcin.2024.06.027>

[20] Wen D, Zhao H, Zhong S, Li C, Liu B, An R, et al. Diagnostic performance of corrected FFR_CT_ metrics to predict hemodynamically significant coronary artery stenosis. Eur Radiol. 2021; 31: 9232-9239. <https://doi.org/10.1007/s00330-021-08064-9>
